# The role of intestinal microbiota in cardiovascular disease

**DOI:** 10.1111/jcmm.14195

**Published:** 2019-02-03

**Authors:** Mengchao Jin, Zhiyuan Qian, Jiayu Yin, Weiting Xu, Xiang Zhou

**Affiliations:** ^1^ Department of Cardiology The Second Affiliated Hospital of Soochow University Suzhou China; ^2^ Department of Neurosurgery The Second Affiliated Hospital of Soochow University Suzhou China

**Keywords:** coronary artery disease, heart failure, hypertension, intestinal microbiota

## Abstract

Accumulating evidence has indicated that intestinal microbiota is involved in the development of various human diseases, including cardiovascular diseases (CVDs). In the recent years, both human and animal experiments have revealed that alterations in the composition and function of intestinal flora, recognized as gut microflora dysbiosis, can accelerate the progression of CVDs. Moreover, intestinal flora metabolizes the diet ingested by the host into a series of metabolites, including trimethylamine N‐oxide, short chain fatty acids, secondary bile acid and indoxyl sulfate, which affects the host physiological processes by activation of numerous signalling pathways. The aim of this review was to summarize the role of gut microbiota in the pathogenesis of CVDs, including coronary artery disease, hypertension and heart failure, which may provide valuable insights into potential therapeutic strategies for CVD that involve interfering with the composition, function and metabolites of the intestinal flora.

## 1 INTRODUCTION

Cardiovascular diseases (CVDs) have become a major health problem because of the associated high morbidity and mortality among patients.^1^, ^2^, ^3^ Over the recent years, the gut microbiota has been found to be linked with multiple diseases, such as obesity,^4^ diabetes mellitus,^5^ digestive system diseases^6^, ^7^ and cancers.^8^ Emerging data indicate the relationship between gut microbiota and CVD.^9^, ^10^, ^11^ There are few microbes in the gut before an infant is born.^12^ The body is constantly stimulated by the outside world from birth onwards and the number of microorganisms in the intestinal tract begins to increase, gradually forming the dynamic balance of the gut microbiota.^13^ When dietary habits, environmental factors, intestinal infection or other factors lead to alterations in the species and quantity of intestinal microorganisms in the adult gut, gut dysbiosis takes place, causing inflammation and metabolic disorders, thus promoting the development of CVD. Bacterial gene sequencing, bioinformatics analysis and metabolomics can help researchers better study the structure and functions of the intestinal microflora and possible signalling pathways that the intestinal microflora may be associated with the development of certain diseases.^12^ In this review, we summarize the roles, mechanisms and therapies associated with the gut microbiota in several common CVDs.

## 2 OVERVIEW OF THE INTESTINAL MICROBIOTA

‘Gut microbiota’ is the umbrella term for the large number of microorganisms that are dependent on the human gut and help the host complete multiple physiological and biochemical functions accompanied by their metabolites. As previously reported, the gastrointestinal tract is known to be home to more bacteria than any other part of the human body.^14^ The composition of the gut microbiota in different anatomical locations of the gut does not change significantly. Eckburg et al performed metagenomic analysis to determine that the gut microbial community consists of six families, namely the Firmicutes, Bacteroidetes, Proteobacteria, Actino‐bacteria, Fusobacteria and Verrucomicrobia phyla, the majority of which are anaerobic organisms.^15^ In the healthy bacterial community, the phyla Firmicutes and Bacteroidetes are the main dominant flora, accounting for more than 90% of the total population.^16^ Unlike the composition of the intestinal microflora, the numbers of microbes residing in different regions of the gut vary greatly. The ascending colon has the highest number of microorganisms, followed by the distal ileum with 10^11^ cells/g and 10^7‐8^ cells/g microorganisms respectively. The content in the proximal ileum and jejunum is less, only 10^2‐3^ cells/g.^17^ The host provides a proper environment and essential nutrients for the intestinal microflora. In turn, the intestinal microflora is involved in the regulation of various human functions, such as providing metabolic nutrition to the host, participating in growth promotion and immune regulation, eliminating pathogenic microorganisms and maintaining the integrity of intestinal barriers and normal homeostasis.^18^ Intestinal microbial ecology can be affected by improper dietary patterns, high stress, life events and use of antibiotics, leading to gut dysbiosis.^12^

## 3 PATHOGENIC ROLE OF INTESTINAL MICROBIOTA IN CVD

### 3.1 Coronary artery disease

The composition and functions of the gut microbiome are affected by external factors that are associated with increased CVD risks, including aging, obesity, a sedentary lifestyle and dietary patterns. In turn, the composition of the gut microbiome may affect the development of CVD.^19^ The observation that DNA from various species of bacteria is found in atherosclerotic lesions and in the gut of the same individuals suggests the gut microbiota may be a potential source of atherosclerotic bacteria and is therefore likely to participate in the pathogenesis of coronary artery disease.^20^, ^21^ Jie et al demonstrated the relationship between the gut microbiota and atherosclerotic CVD.^9^ They identified that the composition of gut microbiome, including members of the *Enterobacteriaceae* and *Streptococcus* spp, was higher in atherosclerotic CVD than in the healthy controls.^9^ Karlsson et al used shotgun sequencing of the gut metagenome to reveal that intestinal microbial communities in patients with symptomatic atherosclerosis were different from those in healthy controls.^22^ Patients had increased numbers of the genus *Collinsella*, while the gender‐ and age‐matched controls had an increased abundance of *Eubacterium* and *Roseburia.* 22 Other evidence in humans also implicated the role of the gut microbiota in the development of atherosclerosis.^23^, ^24^

In addition to these studies in humans, there are a series of animal studies. Chan et al established atherosclerotic models by feeding apolipoprotein E knockout (ApoE−/−) mice a high‐fat diet for 12 weeks supplemented with *Lactobacillus rhamnosus* GG (LGG) or telmisartan (TLM).^25^ Both supplements changed the proportion of the gut microbiota and reduced the atherosclerotic plaque size significantly. Furthermore, five species (*Eubacteria*, *Anaeroplasma*, *Roseburia*, *Oscillospira* and *Dehalobacteria*) have been shown to be effective in preventing atherosclerosis. Similarly, experiments performed by Stepankova et al demonstrated the protective effects of intestinal bacteria on the progression of atherosclerotic lesions.^26^ They fed germ‐free ApoE−/− mice a low‐ or high‐cholesterol diet for 3‐4 months. Atherosclerotic plaques were detected in the aorta of germ‐free mice fed the low‐cholesterol diet. Their study supports the protective effect of bacteria on atherosclerosis. Conversely, Kasahara et al showed that the absence of microbiota could cause an increase in atherosclerotic lesion formation compared with conventionally raised controls.^27^ Other bacteria, including *Porphyromonas gingivalis* and *Aggregatibacter actinomycetemcomitans*, have been validated to be associated with the acceleration of atherosclerosis in animal models after dietary intervention or intravenous infusion.^28^, ^29^, ^30^

In the light of these findings, some types of gut bacteria have been identified as novel contributors to the progression of atherosclerosis, while others can protect against atherosclerotic plaque lesions. It is still unknown how the microbes that reside within our bodies can drive or initiate the development of atherosclerosis. It is unclear which species play a leading role in contributing to CVD and the detailed mechanisms involved require further investigation.

One microbial metabolite, trimethylamine N‐oxide (TMAO), has gained considerable attention as a major influencing factor in CVD. The composition of the gut microbiota is altered when dietary patterns change. Trimethylamine (TMA) is generated by the altered microbiota through metabolizing choline,^31^ phosphatidylcholine,^32^ L‐carnitine^33^ and betaine^34^ via a range of microbial enzymes, primarily TMA lyases. Then TMA enters the liver through the portal circulation and is oxidized into TMAO by hepatic flavin monooxygenases (FMO3).^31^, ^35^, ^36^ Wang et al fed C57BL/6J mice a choline‐enhanced diet and ApoE−/− mice showed an increased risk of atherosclerosis.^31^ Furthermore, up‐regulation of some types of macrophage scavenger receptors and atherosclerosis was promoted after supplementing mice with TMAO. The serum TMAO level was also positively correlated with the atherosclerotic plaque area. This may be explained by the role of TMAO in inhibiting reverse cholesterol transport and accumulating macrophage cholesterol by means of inducing cell surface expression of two scavenger receptors (differentiation 36 and scavenger receptor A).^31^, ^33^, ^37^ As a result, increased foam cell formation accelerates atherosclerosis. Additionally, TMAO can decrease cholesterol clearance from the body via inhibiting bile acid synthesis.^38^, ^39^ However, conversely, one study found that TMAO could reduce cholesterol reabsorption and protect against atherosclerosis formation.^40^

Recently, a series of clinical trials showed the relationship between gut microbiota and CVD events. A study by Li et al revealed that the TMAO level in acute coronary syndromes was an independent predictor of both short‐term (30 days and 6 months) and long‐term (7‐year) major adverse cardiac events.^41^ Other studies have also highlighted the participation of TMAO in the development of CVD in a variety of patient cohorts.^42^, ^43^, ^44^ Collectively, mounting evidence suggests that TMAO is part of an important mechanism by which the intestinal microflora influence CVD.

### 3.2 Hypertension

The gut microbiota consists of four major phyla: Firmicutes, Bacteroidetes, Actinobacteria and Proteobacteria. Firmicutes and Bacteroidetes account for a large part of the intestinal microflora. The ratio of Firmicutes (F) and Bacteroidetes (B) (F/B) is considered a biomarker for gut dysbiosis.^45^ Yang et al demonstrated that microbial richness, diversity and evenness were decreased not only in spontaneously hypertensive rat models but also in a cohort of patients with high blood pressure.^46^ Additionally, an increased F/B ratio and decreased numbers of acetate‐ and butyrate‐producing bacteria were observed. In Ang II‐infused rats, minocycline intervention was able to lower the blood pressure and induce changes such as increased gut microbial diversity, decreased F/B ratio and expanded populations of acetate‐ and butyrate‐producing bacteria. This indicates that hypertension is linked to gut dysbiosis and that improving gut microbiota may be a target for future therapies for hypertension. Adnan et al^10^ found that blood pressure can be altered through exchanging the gut microbiota between spontaneously hypertension/stroke‐prone rats (SHRSP) and Wistar‐Kyoto rats (WKY). Systolic blood pressure and F/B ratio were both increased in WKY rats after gavage with SHRSP microbiota. Conversely, systolic blood pressure was decreased in SHRSP rats after gavage with WKY microbiota, although this was not statistically significant. Similarly, the importance of gut microbiota in hypertension formation is evident from a study showing elevated blood pressure in germ‐free mice after transferring faecal material from hypertensive patients to the mice.^47^ A recent study demonstrated that there were more opportunistic pathogenic taxa (*Klebsiella* spp, *Streptococcus* spp and *Parabacteroides merdae*) involved in the pathogenesis of hypertension and that these were related to the severity of disease.^48^ These data suggested the direct and indirect role of gut microbiota in the development of hypertension. A number of experiments have further investigated the causality relationship between gut microbiota and high blood pressure in the recent years.

The mechanisms of hypertension are complex and multifactorial. The gut microbiota is considered a risk factor for a variety of diseases including atherosclerosis,^23^ obesity,^49^ metabolic syndrome^50^ and diabetes,^51^ which are proven to be connected with hypertension. In comparison with other intestinal microflora metabolites, more studies have shown the physiological functions of short chain fatty acids (SCFAs) in blood pressure regulation. Bacteria in the gut produce SCFAs by anaerobic fermentation of dietary fibre. The most common SCFAs are acetate, propionate and butyrate. SCFAs are known to mediate multiple biological effects on the endocrine system, the nervous system, CVD, inflammation and gut homeostasis via binding to their receptors, which include G protein‐coupled receptor 41 (GPR41), G protein‐coupled receptor 43 (GPR43), G protein‐coupled receptor 109A (GPR 109A) and vascular olfactory receptor 78 (Olfr78).^52^, ^53^ Olfr78 is expressed in olfactory neurons, renal afferent arterioles as well as in vascular smooth muscle cells, where it plays a role in blood pressure regulation.^54^, ^55^, ^56^ Olfr78 was found to elevate renin levels, resulting in increased blood pressure, while GPR41 had an antagonistic effect.^55^ Pluznick et al treated Olfr78^˗/˗ ^and wild‐type mice with antibiotics for a set time period to determine whether metabolites from the microbiota mediate blood pressure via Olfr78.^55^ The results showed that blood pressure was increased in Olfr78^‐/‐ ^mice after antibiotic treatment, but no effect was seen in the control mice. This indicated that Olfr78 contributes to the hypertensive effects by means of SCFAs. Pluznick et al indicated that GPR41 and Olfr78 had opposing functions in the modulation of blood pressure after responding to propionate (a type of SCFA).^56^ In addition, SCFAs have also been shown to induce vasorelaxation,^57^ while other studies have revealed that GRP41 appears to decrease cAMP levels through Gαi.^58^, ^59^

Thus, blood pressure is closely linked to the diversity, richness and evenness of the microbiome living in the gut and it is affected by the F/B ratio. The hypertensive and hypotensive effects of SCFAs are mediated by binding to Olfr78 and GPR41 respectively. The discovery that drug intervention has an influence on blood pressure modulation by changing the species as well as the metabolites of the gut microbiota may provide new ideas for the treatment of hypertension. Moreover, how the intestinal microflora influences blood pressure deserves further exploration.

### 3.3 Heart failure

Heart failure (HF) encompasses a group of complex clinical syndromes that result in damage to the structure or function of the heart. HF is the end stage of many CVDs. Although pharmacological and non‐pharmacological therapies can delay the progression of HF, the short‐ and long‐term mortality rates remain high.^60^, ^61^, ^62^ Our understanding of the pathophysiological mechanisms of HF has changed greatly in the recent years. The main concept shift is from haemodynamic changes to neurohumoural‐immune regulation. The role of gut microbiota in inflammatory and immune response has drawn attention to the link between the gut microflora and HF.^63^, ^64^

Pasini et al compared the bacteria and fungi in the faeces of HF patients with those of healthy controls.^11^ The results showed that patients with chronic heart failure (CHF) were colonized by more pathogenic bacteria than the control patients. *Candida*, *Campylobacter* and *Shigella* species were proven to be positively correlated with the severity of disease. Compared with healthy controls, intestinal permeability (IP) increased for 78.3% of the patients with CHF. The gut was more permeable in patients with moderate and severe CHF than patients with mild CHF. Right atrial pressure was positively correlated with IP. In another animal experiment, the abundance of 10 types of faecal flora was changed in HF guinea pigs with pressure overload.^65^ These data suggest that HF can disrupt the balance of intestinal microflora. This prompted researchers to propose the ‘gut hypothesis’. Decreased cardiac output, leading to low perfusion and gastrointestinal congestion, can induce intestinal ischaemia and/or oedema in patients with HF. As a result, the composition of the gut microbiota, intestinal function, morphology and IP are all altered. Secondary intestinal bacterial translocation and increased levels of circulating endotoxin accelerate the systemic inflammatory response, while the activated inflammatory cytokines contribute to HF.^66^, ^67^, ^68^

Collectively, changes in the intestinal microflora exist in patients with HF. The aforementioned metabolite TMAO generated by the gut microbiota has a certain significance in HF patients. Two cohort studies, which enrolled hundreds of participants, demonstrated that elevated TMAO levels were predictive of the long‐term mortality risk in patients suffering from not only CHF,^69^ but also acute HF.^70^ TMAO is likely to provide a basis for risk stratification of HF. Organ et al used transverse aortic constriction surgery to induce HF in C57BL6/J mice and found that in mice fed with either TMAO or choline supplemented diets, worse symptoms and signs of HF were observed compared with mice fed a control diet.^66^ Additionally, plasma levels of TMAO increased in mice fed with dietary TMAO as well as choline because of conversion of choline to TMA by gut microbes. TMAO could accelerate the development of left ventricular dilation, myocardial fibrosis and ventricular remodelling. In agreement with Organ's observations, Li et al also demonstrated that TMAO played a role in the development of cardiac hypertrophy and cardiac fibrosis.^71^ The mechanism of increased circulating TMAO levels in patients with HF remains to be determined. Some other gut‐derived metabolites have also been shown to have an impact on HF. Secondary bile acid, transformed by the gut microbiota, was reported to increase in CHF patients,^64^ and indoxyl sulfate has been linked with myocardial fibrosis and ventricular remodelling.^72^

In addition to gut microbiota metabolites mentioned above, p‐cresyl sulfate (PCS) and phenylacetylglutamine (PAG) are involved in CVDs as well.^73^, ^74^ PCS is a component of phenolic end products generated by gut microorganism via metabolizing aromatic amino acids, like tyrosine and phenylalanine, in the intestine.^75^ PCS levels have been shown to predict cardiovascular events and all‐cause mortality in elderly haemodialysis patients.^73^ Likewise, PAG is one of the colonic microbial metabolites produced by glutamine conjugation of phenylacetic acid, high levels of which were known as a strong and independent risk factor for CVD and mortality in patients with chronic kidney disease.^74^

## 4 THERAPEUTICS BASED ON THE MICROBIOTA

Recent studies have shown that intestinal microbiota is critically involved in cardiovascular health and diseases.^76^, ^77^ For the treatment of CVD, researchers have gradually turned their attention to the intestinal microflora and related metabolites. Consequently, the gut microbiome, as a novel regulator of CVD, has become a potential target for therapeutics.

### 4.1 Antibiotics

Broad‐spectrum antibiotics are commonly used in cardiovascular experiments targeting the gut microbiota. Galla et al administered three types of oral antibiotics (neomycin, minocycline and vancomycin) to Dahl salt‐sensitive (S) rats and spontaneously hypertensive rats (SHR) to investigate any changes in blood pressure. They found alterations in the intestinal microflora accompanied by increased systolic blood pressure in the S rats and decreased systolic blood pressure in the SHR after minocycline and vancomycin intervention, but not neomycin intervention.^78^ Rune et al found that ampicillin could reduce LDL and VLDL cholesterol levels, which were risk factors for atherosclerosis in the mice model, leading to decreased aortic atherosclerotic lesion areas.^79^ In trials on patients, whether the use of antibiotics has protective effects against CVD remains unanswered. Some studies showed beneficial effects,^71^ while others did not.^80^ Therefore, strategies for treating CVD with antibiotics remain controversial, because broad‐spectrum antibiotics mediate a wide range of effects. Thus, the potential benefits of antibiotics need to be weighed against the potential side effects.

### 4.2 Faecal microbiota transplantation

Faecal microbiota transplantation (FMT) is capable of contributing nutrients, inhibiting the growth of pathogenic bacteria and regulating the immune system of the host through transplanting functional bacteria from healthy individuals into the gastrointestinal tract of patients, thereby helping patients reconstruct the normal functions of the gut microbiota.^81^ FMT is effective in treating recurrent *Clostridium difficile* infection,^82^ inflammatory bowel disease^83^ and irritable bowel syndrome.^84^ However, the potential for treating CVD needs further investigation. In a double‐blind randomized controlled pilot study, the composition of the gut microbiota in metabolic syndrome patients changed significantly after transplantation of vegan‐donor faecal microbiota.^85^ However, there was no change in the gut‐derived metabolite TMAO. As mentioned above, germ‐free mice showed increased blood pressure following FMT from patients with hypertension.^47^ For now, FMT remains a promising therapy for CVD, although more studies are needed.

### 4.3 Probiotics and prebiotics

Proper supplementation with probiotics is able to interfere with the functions and composition of intestinal microflora, resulting in activation of the immune system and control of inflammation. Common probiotics include *Lactobacillus*, *Bifidobacterium*, *Lactococcus*, *Streptococcus* and *Enterococcus*.^86^ Prebiotics can stimulate the growth and/or activity of one or more beneficial bacteria selectively and have beneficial effects on the host. Most prebiotics are carbohydrates that are present in natural products such as fruit, vegetables and cereals.^86^ The utility of probiotics and prebiotics in changing the intestinal microecology has become a novel target for the prevention and treatment of CVD. In one study, researchers fed ApoE^−/−^ mice different types of inulin (a kind of prebiotic) to observe the relationship between inulin and atherosclerosis. The results showed that atherosclerotic lesions decreased by 35% and 25% after long‐chain inulin intervention and oligofructose‐enriched inulin supplementation, respectively, compared with mice fed a control diet. A number of studies have shown that both probiotics and prebiotics have beneficial effects on lipid modulation,^87^, ^88^, ^89^, ^90^ and thereby indirect benefits on CVD. Thus, patients with CVD can supplement their diet with probiotics and prebiotics according to their own condition to regulate the gut microbiota and improve gut dysbiosis.

### 4.4 Dietary intervention

Several studies have examined the effects of diet on the gut flora and disease by administering mice a high‐salt diet (HSD).^91^, ^92^, ^93^ Bier et al investigated the relationship between a HSD and microbial variation, as well as metabolite levels.^91^ The composition of the gut microbiota accompanied by SCFAs was shown to be altered in the HSD‐fed mice, inducing hypertension. Three genera including the members of the families Erwinia, Christensenellaceae and Corynebacteriaceae were significantly increased in the HSD‐fed mice compared with mice fed the control diet, while a reduction in the genera Anaerostipes was also observed in the HSD‐fed mice. Moreover, seven taxa were found to be associated with blood pressure. Marques et al treated mice with a high‐fibre diet or a diet supplemented with acetate, leading to a prominent alteration in gut microbes and elevation of SCFAs levels, which had a protective effect on hypertension and HF.^94^ Furthermore, resveratrol, found in grapes and berries, had favourable effects on atherosclerosis via attenuating TMAO by inhibiting TMA generation.^95^ Thus, it is important for patients to make adjustments for their diet to delay the progression of CVD.

## 5 OTHER THERAPIES

Some research has been conducted on protecting cardiovascular system with chemicals that inhibit microbial metabolic processes. For example, 3, 3‐dimethyl‐1‐butanol, a non‐lethal inhibitor of TMA formation, reduced atherosclerotic lesions by decreasing the levels of TMAO that were converted from TMA in ApoE−/− mice.^96^ To the best of our knowledge, TMA is oxidized into TMAO by hepatic FMO3. Therefore, some studies have focused on chemical substances that could act as potential FMO3 inhibitors to prevent or treat atherosclerosis.^97^, ^98^, ^99^ Gao et al found that the binding of methimazole and indole could provide evidence for the development of human FMO3 inhibitors.^97^ Indole‐3‐carbinol (I3C) and its acid condensation products, I33’ and LT, were reportedly responsible for the inhibitory activity of human FMO3.^98^ Therapeutically, the aim was to develop targeted drugs for the intervention of certain CVDs.

## 6 SUMMARY

**Figure 1:**
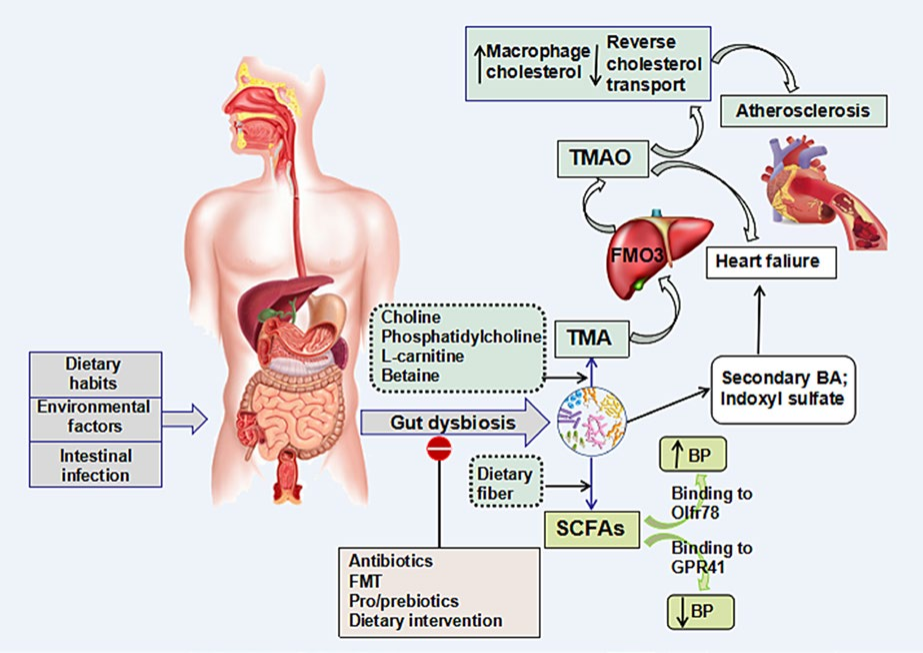
The role of intestinal microbiota in cardiovascular diseases. Dietary habits, environmental factors and intestinal infection can alter the gut microbiota from eubiosis to dysbiosis. The intestinal microbiota metabolizes choline, phosphatidylcholine, L‐carnitine and betaine, generating trimethylamine (TMA), which is oxidized into trimethylamine N‐oxide (TMAO) by hepatic flavin monooxygenases (FMO3). TMAO can accelerate atherosclerosis by inhibiting reverse cholesterol transport and accumulating macrophage cholesterol. Other intestinal microflora metabolite, short chain fatty acids (SCFAs), regulate blood pressure by combining with Olfr78 and GPR41. Secondary bile acid and indoxyl sulfate are associated with heart failure. Specific treatments including antibiotics, faecal microbiota transplantation (FMT), pro/prebiotics and dietary intervention can improve gut dysbiosis

Trillions of bacteria reside in the human gut, mainly divided into probiotics, neutral bacteria and pathogenic bacteria. Among them, the Firmicutes and Bacteroidetes families occupy the highest proportion and the F/B ratio is a vital parameter reflecting intestinal disorders. Factors such as lifestyle, drug application and intestinal environment disorders may contribute to gut dysbiosis, promoting the occurrence and development of CVD. Changes in the gut microbiota of individuals with coronary artery disease, hypertension and HF have been observed in both human and animal experiments, but the specific mechanisms are still under study (Figure 1). Research has demonstrated that the elevation of TMAO was associated not only with the development of atherosclerotic plaques, but also with the poor prognosis of patients with HF. SCFAs can regulate blood pressure by combining with Olfr78 and GPR41.

Because the gut microbiota is closely related to CVD, new ways to treat CVD based on the gut microbiota are emerging. For patients with CVD, reasonable adjustment of diet structure and optimization of diet composition are particularly important for maintaining physical health. Prebiotics can promote the growth of probiotics and inhibit the growth of pathogen. Supplement of prebiotics and/or probiotics may be effective and feasible in the treatment of CVD. FMT is limited mainly because it causes great changes in the intestinal microflora of the patients, and at the same time, it brings both beneficial and harmful bacteria to the patients, which may lead to adverse complications.^100^, ^101^ The use of targeted drugs and antibiotics is controversial because of adverse drug reactions.

In the future, more experiments will be conducted to investigate the correlation between the gut microbiota and CVD. The direction will focus on the exploration of intestinal bacteria directly related to the occurrence and development of CVD. The pathogenesis of intestinal flora and its metabolites is not fully understood and the relevant basic and clinical studies need to be further carried out. Some new microecological preparation can ameliorate CVD through improvement of gut microbiota,^102^ the research and development of which are still in the clinical trial stage. The potential benefits and limitations of microecological preparation in the treatment of CVD need more work. Strategies to improve the intestinal microbiota are expected to be new approaches for the treatment of CVD.

## CONFLICTS OF INTEREST

The authors declare that there are no conflicts of interest.
